# Plant Species Diversity and Composition Differ Significantly Between the Boundaries of Kettle Holes and Field Borders

**DOI:** 10.1002/ece3.70515

**Published:** 2024-11-07

**Authors:** I. Heyer, M. Wulf

**Affiliations:** ^1^ Centre for Agricultural Landscape Research (ZALF) Müncheberg Germany; ^2^ Institute of Biochemistry and Biology, University of Potsdam Potsdam Germany

**Keywords:** arable cropping systems, crop edge, field margin, Hill numbers, kettle holes, PERMANOVA, vegetation analysis

## Abstract

The boundaries around habitat islands in agricultural fields are rather unexamined, although they may be an important part of agroecosystems in some regions. In this study, we surveyed field boundaries in northeastern Brandenburg both at outer field borders and around kettle holes, which are typical habitat islands in the region. We examined, described, and compared the plant species diversity and composition at both the inner and outer field boundaries in the arable fields (crop edge) as well as in permanent vegetation (field margins). Diversity was assessed and compared with Hill diversity values, using the iNext framework. Non‐metric multidimensional scaling and permutational analysis of variance were used to compare species composition at different field boundaries and to search for variables that drive species composition at the local scale. The results revealed that both species diversity and composition differed significantly between the inner and outer boundary along the crop edges and at the field margins. Local site conditions, namely a moisture gradient, influenced the species composition of the field margins, resulting in differences between the inner and outer field margins. Mitigated through crop growth and cover, the moisture gradient influenced also the species composition of the inner and outer crop edges, despite the management practices on the fields were the same.

## Graphical abstract

Plant species of field boundaries around kettle holes in arable fields were surveyed. These inner field boundaries are significantly different in both, plant species richness and composition, compared to outer field boundaries.

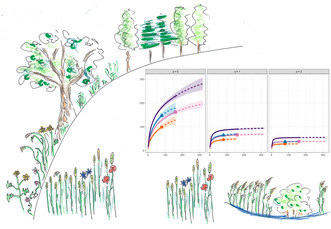

## 1 Introduction

### 1.1 Field Boundaries

Plants constitute the basis of food webs, providing several resources for higher trophic level organisms in ecosystems, e.g., pollen, nectar and prey, shelter and habitats for nesting and overwintering (Bàrberi et al. 2010; Bürki and Pfiffner 2000; Thies, Denys, and Tscharntke 2000). In arable agricultural systems, these functions are largely provided by so‐called “weeds”, i.e. plants other than crops growing in cropped areas (Marshall et al. 2003). Spontaneous vegetation at the field margins can also colonize cropped fields and is then also considered weeds (Marshall and Arnold 1995). These plants contribute to the diversity of vegetation in agricultural landscapes (Marshall and Moonen 2002). In landscapes with intensively managed agricultural areas with large fields, many of the formerly diverse small habitat patches have been lost in recent decades, and field margin strips are often the last remaining diverse features (Baessler and Klotz 2006). Given the decline in biodiversity in agricultural landscapes (Jandt et al. 2022; Billeter et al. 2008; Krebs et al. 1999), field boundaries play an important role as refuges for species across several trophic levels (Meyer et al. 2015; Batáry et al. 2017; Warzecha et al. 2018; Douglas, Vickery, and Benton 2009).

**FIGURE 1:**
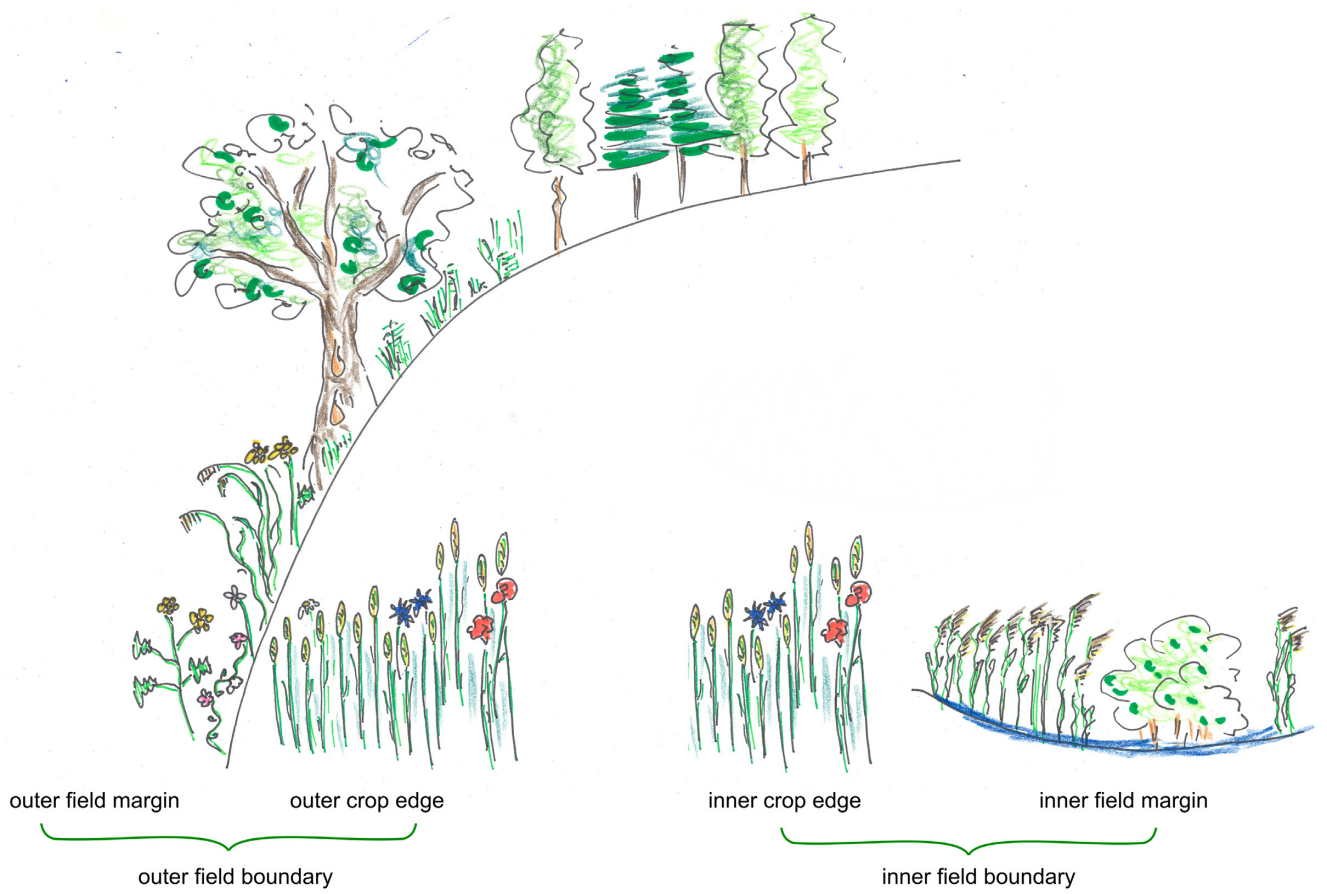
Compartments of the field boundary and terms used. ©Ines Heyer.

Field boundaries can have highly variable characteristics and functions, and for that reason, the terminology used differs. Here, the term “field boundary” describes the borders of arable fields, i.e. the areas on the outsides of fields as well as the borders of habitat islands inside fields. Field boundaries consist of two compartments: the crop edge (CE), which is defined as the outer part of the arable field and is cropped and managed by farmers (Figure 1), and the field margin (FM), which is defined as the permanent vegetation outside the arable field and all structures that can be present there (hedges, trees, walls, ditches, etc.) (Figure 1).

The decrease in species richness from the edges of areas into the interior has been well‐known since decades and has been highlighted by many studies (Wilson and Aebischer 1995; Batáry et al. 2017; Seifert, Leuschner, and Culmsee 2015; Seifert et al. 2014; Nagy et al. 2018; Štefanić et al. 2020; Wietzke et al. 2020). Crop edges are often less favorable for crop growth, therefore, there are more opportunities for weeds to occupy this space, for example, due to higher light availability (Seifert et al. 2014; Wietzke et al. 2020). This heterogeneity results from lower fertilizer and herbicide inputs (Wilson and Aebischer 1995) or, more generally, less disturbance by farming (Fried et al. 2009). Crop edges can also harbor species from adjacent habitats, at least in the first meters of the cropped area (Marshall 1989; Fried et al. 2009; Wietzke et al. 2020). Several species can cope with the conditions on both sides of these transition zones (Marshall and Arnold 1995), and perennial plants from margins frequently grow at crop edges (Fried et al. 2009; Gabriel et al. 2006).

In the field margins, heterogeneity in plant species composition is strongly related to the heterogeneity of margin structures. Aavik et al. (2008) reported that small‐scale landscape features, namely, the presence or absence of trees, ditches, or roads, within a radius of 10 m around the plots significantly influence species composition. In particular, the presence of trees or shrubs, in contrast to open, grassy boundaries, has been observed in several studies (Le Coeur, Baudry, and Burel 1997, Blaix and Moonen 2020, Aavik and Liira 2010). This contrast between margins can be described as a gradient from moist and shady habitats to open and dry habitats. Considering only the dryer grassy boundary structures, the management of the boundary plays a significant role in determining the plant species composition (Hovd and Skogen 2005; Blaix and Moonen 2020).

### 1.2 Habitat Islands

Small habitats in agricultural landscapes can be located like islands within a field—especially in large cropped areas. Embedded in the matrix of agricultural land, habitat islands are well‐studied habitats in agricultural landscapes and it is widely known that they provide important functions, biotopes, and refuges for several groups of organisms (Lindborg et al. 2014; Riggi and Berggren 2020; Cousins 2006; Cousins and Lindborg 2008). These areas can act as stepping stones if they are not too isolated (Vasić et al. 2020; Cousins 2006; Cousins and Lindborg 2008), or harbor species that are not present in the agricultural matrix (Schöpke et al. 2019). Habitat islands can serve as refuges for plant species in semi‐natural grasslands, especially in agricultural landscapes with greater habitat fragmentation (Deák et al. 2020; Lindborg et al. 2014).

Kettle holes are a unique type of habitat island that are distributed across the Northern Hemisphere and often coincide with agricultural areas (Vasić et al. 2020). They are defined as “…small depressions formed by the melting of remnant ice blocks that are permanently or periodically filled with water” (Vasić et al. 2020). In intensively used landscapes, they are important for maintaining ecosystem functioning since they provide habitats for many groups of organisms, such as plants and pollinators, can be stepping stones in croplands and influence the hydrological balance and temperature of the surrounding areas (Vasić et al. 2020). Kettle holes can be very variable in size, depth, and water level, and these characteristics are also seasonally variable; thus they can harbor very heterogeneous plant communities. Species turnover between these habitats can be very high (Kalettka and Rudat 2006; Schöpke et al. 2019). In the moraine landscapes of northern Germany, kettle holes are the dominant type of habitat islands. In some regions, 40 or more kettle holes can be found per square km (Kalettka and Rudat 2006, Schöpke et al. 2019). Presuming that the presence and length of edges and the amount of habitat in landscapes are crucial for diversity at field boundaries, it is logical to conclude “…that midfield islets are valuable sources of diversity in the landscape” (Wrzesien and Denisow 2016).

### 1.3 Aims and Objectives

The aim of our study was to describe and examine the diversity and composition of plant communities at inner field boundaries around kettle holes. We did this for the two boundary compartments, CE and FM, and compared the inner boundaries around kettle holes with the outer boundaries. In particular, we tested the following hypotheses:

- The vegetation composition and plant species diversity of the CEs at the inner and outer field boundaries are not significantly different.
- The vegetation composition and plant species diversity of the FM at the inner and outer field boundaries are significantly different.
- The factors that drive vegetation composition in the CE and FM are significantly different. Moreover, (1) we expect a strong effect of crop abundance and height on the vegetation composition in the CE, whereas (2) we expect local field margin factors to strongly affect the species composition in the permanent FM.

## 2 Methods

### 2.1 Study Region

**FIGURE 2:**
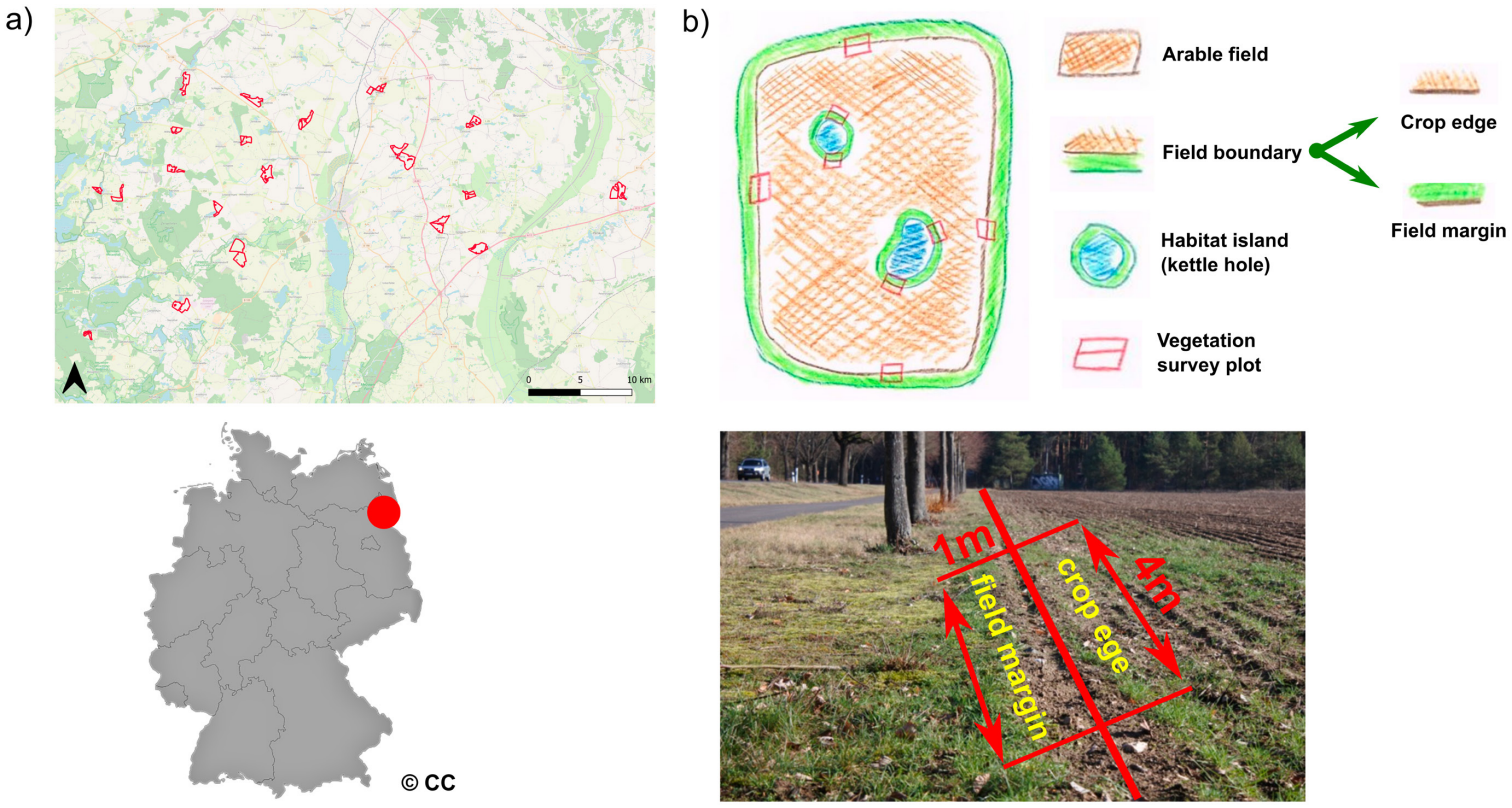
Study region in north‐eastern Germany and distribution of surveyed field pairs in the study region (a) and representative vegetation survey design for a field with kettle holes as well as the location of the plots on the field boundary (b). Fields without kettle holes contain only survey plots on the outer boundaries. The map of Germany is from Wikipedia and was created by DeStatis; it is used under CC BY‐SA 2.0 and modified by the authors. The background map of the field distribution was obtained from OSM and is used under CC BY‐SA 2.0.

Field sampling was carried out in the Uckermark (NE Germany) around the city of Prenzlau (Figure 2). The Uckermark is characterized by a temperate climate. The mean annual temperature is 8.9°C, and the mean annual rainfall is 521 mm (station Angermünde, long‐time means 1981–2010; DWD 2023). The soils and landscapes in this region are formed by Weichsel‐glaciation (duration from approximately 11,500 to about 10,200 years ago). The substrate of the moraine landscape is loamy and sandy, including mainly base‐rich soils with high permeability (LBGR—Landesamt für Bergbau 2023). These soils are referred to as Aerosols, Luvisols, and Retisols according to the international soil classification system (World Reference Base for Soil Resources—WRB 2023). Arable soil qualities (in German “Ackerzahl,” which can be described as agricultural yield score) vary between 33 and 64 (with 100 being the highest possible number for fertile brown earth), which is above the average for Brandenburg (Ministerium Für Landwirtschaft 2023). The landscape is rolling and dominated by arable fields with characteristic elements such as kettle holes (Schlaak 1999).

We selected 40 fields, all of which were managed conventionally and cultivated with winter cereals during the year of the survey. The fields were chosen in pairs, with each pair containing one field without any habitat islands and the other field having at least one habitat island. As kettle holes are the dominant type of habitat islands in this region, only kettle holes were considered when identifying habitat islands. The fields containing each pair were located near each other but separated by a physical barrier such as a field path, a lane, or a hedge—they were not allowed to border each other directly. The field pairs were separated by a distance of at least 2.5 km (measured from field pair centroids) to prevent spatial autocorrelations.

### 2.2 Field Survey

In each field, the vegetation of the field boundary was surveyed once, between the end of May and the end of June in the years 2021 and 2022. In total, 40 fields were sampled, with 18 being sampled in 2021 and 22 in 2022. Fields had an average size of 49.6 ha. Four vegetation survey plots were located on the outer field boundary, and an additional four plots were located on the inner field boundaries around the kettle holes in each field with habitat islands (Figure 2).

To place the outer plots, the perimeter of each field was divided into four equal parts, and the plots were placed at the divisions.

Kettle holes in the surveyed fields had a mean size of 0.54 ha (min 0.02, max 2.77). If there was more than one kettle hole in a field, the inner plots were partitioned between them to obtain as much insight as possible into the heterogeneity of the kettle holes boundary vegetation.

Each plot was 2 × 4 m in size and was separated into the CE on the arable field and the FM, which was characterized by permanent vegetation. The outermost crop row was defined as the border between these two parts of the field boundary (Wietzke and Leuschner 2020). Therefore, on each side of the border, an area of 1 × 4 m was surveyed. For the most accurate estimation of plant abundance, the plots were divided into 1 m^2^ subplots. In the subplots, all the plant species, their life stages (seedlings, juveniles, fully developed herbs), and their abundances were recorded. To estimate the abundances of the plants the decimal scale from Londo was used (Londo 1976). This decimal scale is comparable to that of Braun‐Blanquet but with finer levels, with which percentage cover and number of individuals can be combined (see also Alberdi, Condés, and Martínez‐Millán 2010). The cover and height of the vegetation (average height of the shortest and tallest herbs as well as the maximum herb height) were also recorded for each subplot. The vegetation data were stored in the TurboVeg database for Windows 2.151b (Hennekens and Schaminee 2001), with one database entry for each square meter. In total, 1920 subplots were recorded (20 field pairs × 12 plots × 8 subplots).

### 2.3 Location and Site Variables

Each plot was described by its location variables: name of the field pair, field with or without a kettle hole, and FM or CE on the inner or outer boundary (Table 1). Additionally, in each plot, the height of the plants and cover of the plants, litter, and bare soil (percentages) were estimated.

**TABLE 1:** Plot information, boundary categories and cover and height values of herbs and crops with their distribution levels, mean values for inner and outer boundaries, and tests of differences between inner and outer boundaries. Levels of significance: ***p<0.001; **p<0.01; +*p*<0.05.

Plot information									
**Descriptor**		**Level**			**Crop type**	**Nr of plots**	**Nr of fields**		
Field pair		20 names			Spelt	24	2		
Kettle hole		Yes/No			Barley	56	6		
Field position		Inner/Outer boundary			Rye	64	1		
Boundary position		CE/FM			Triticale	16	5		
					Wheat	320	26		
**Boundary categories**									
**All categorical with two levels**				**0**	**1**	**Percent 0/1**	**Chi‐square *p* value (difference between inner and outer boundaries)**		
Mowed				431	46	85/14	0.000***		
Water				407	70	85/14	0.001***		
Hedge				392	85	82/17	0.001***		
Trees				224	253	46/53	0.299		
Forest				453	24	95/5	Boundary categories with less than 10% occurrences in one level were not considered for further analysis		
Grassy				26	451	95/5			
Grassland				443	34	92/7			
Road				336	141	70/30	0.000***		
**Cover and height values**									
	**Unit**	**Value**	**Mean inner CE**	**Mean outer CE**	**Wilcoxon rank test *p* value (difference between inner and outer boundaries)**		**Mean inner FM**	**Mean outer FM**	**Wilcoxon rank test *p* value (difference between inner and outer boundaries)**
Cov_herbs	%	0–98.25	13.94	12.45	0.879		74.39	69.56	0.041*
Herb_high	cm	0–140	51.76	43.23	0.074		73.8	66.45	0.02*
Cov_crop	%	0–98.75	75.88	66.78	0.000***				
Crop_high	cm	0–193.75	91.62	82.84	0.010**				

In addition, boundary categories on the outer and inner boundaries were recorded, within a radius of 10 m around the plot. These categories included adjacent boundary features such as roads, water, hedges, shadowing from trees, and management, which reflected mowing of the field margin vegetation (Table 1). For the kettle holes, in addition to the boundary categories, the dominant vegetation type of the whole kettle hole was recorded (trees, shrubs, or herbaceous plants) and the presence or absence of water was noted. For subplots on the CE, the crop type, crop cover, and crop height were recorded. Owing to limited resources (time and funding), no soil analyses could be performed.

### 2.4 Data Analyses

All data analyses were conducted using R version 4.2.2 (R Core Team 2022). The package *vegdata* (Jansen and Dengler 2010) was used for loading the TurboVeg data, harmonizing the taxonomy, transforming the Londo scale into percentage cover values, and building several working tables for further analysis. For the analysis, the eight subplots of each survey plot were aggregated into 480 plots with mean values for abundance, height, and cover. The *vegan* package, version 2.6‐4 (Oksanen et al. 2022), was used for NMDS and PERMANOVA and the *iNEXT4*.*steps* package (Chao and Hu 2020) was used to analyze the species diversity.

#### 2.4.1 Species Diversity

In four steps, the Hill diversities of the inner and outer FMs as well as inner and outer CEs were examined to compare the diversities of the different assemblages (Chao and Hu 2020). When calculating Hill diversities, it is possible to model the influence of common or rare species via the order of the exponent *q* in the formula (Chao et al. 2014). By transferring commonly used diversity indices into true diversities, effective numbers of species are obtained, a measure that accounts for species richness as well as the relative abundances of different species (Chao, Chiu, and Jost 2016). While a Hill diversity of order *q* = 0 counts all species equally, but ignores their abundances and thus gives numbers for species richness, orders *q* = 1 and *q* = 2 take the abundances into account. Order *q* = 1 is equivalent to Shannon diversity, and order *q* = 2 is equivalent to Simpsons diversity (Hsieh, Ma, and Chao 2016). The latter order focuses on the highly dominant species, while the Shannon index‐equivalent counts species in relation to their individual abundances, so it can be interpreted as the diversity of common species (Chao et al. 2020).

All four steps were conducted for the three commonly used orders of *q* = 0, *q* = 1, and *q* = 2. Four‐step analysis was used to extend the analysis of the Hill diversities of different assemblages via rarefaction and extrapolation, as suggested by Chao et al. (2014) and Hsieh, Ma, and Chao (2016). In addition to comparing assemblages on the basis of their sample sizes and their sample coverages, the four‐step analysis also considers sample completeness and sample evenness. In all steps, the plotted curves were calculated via rarefaction up to the observed assemblage diversity and were further extrapolated to double the sample size. The analysis included a sample coverage‐based non‐asymptotic plot of assemblage diversities which made it possible to compare assemblages or diversities with unequal sample completeness. This approach should be used if the asymptotic diversity curves do not reach the asymptote, even not in the extrapolated area of the curve, which is often the case for species richness, so *q* = 0. With the non‐asymptotic curves, the diversity of the assemblages can be compared at a calculated maximum sample coverage value of *C*_max_. With this calculated maximum sample coverage value, all the samples were compared at the same sample completeness, and the diversity was plotted as a function of sample coverage up to that point. Evenness was also calculated at the *C*_max_‐value to make it comparable, although the species richness of the samples was not the same. All curves were calculated with a 95% confidence interval, which was obtained by a bootstrap method (Chao et al. 2020). For evenness, the Pielou index was used, this index ranges between 0 and 1 with values closer to 1 indicating communities that are more even (Pielou 1966).

#### 2.4.2 Species Composition and Explanatory Factors

To gain insight into the community composition of the different boundary vegetation types, we used non‐metric multidimensional scaling (NMDS), which orders plots according to similarities in their species composition in reduced ordination space (Legendre and Legendre 1998). We used abundance data with Bray–Curtis distances as dissimilarity measures. Three rows, which constitute 0.6% of the whole dataset, without any vegetation data (all on the CE) were removed before analysis. Two subsets for the CE and FM vegetation were created and analyzed separately to accept or reject the community composition parts of hypotheses (i) and (ii). For both subsets, NMDS was conducted with four dimensions and 100 maximum trials. The data were standardized with Wisconsin double standardization (to equalize the weights of samples and species between 0 and 1) and square root transformed during the analysis. The positions of the plots on the fields (inner or outer boundary) were subsequently fitted to the NMDS. To visualize whether the group centroids of the inner and outer FM and the inner and outer CE were separate from each other with respect to their species compositions, the survey plots were associated with the centroids for the inner and outer FM and CE.

To test whether the group centroids of the inner and outer CE and the inner and outer FM were significantly different, we used permutational multivariate analysis of variance (PERMANOVA) according to the description by Anderson (2017). Before performing PERMANOVA, we conducted a test to assess the presence of equal multivariate dispersion of groups due to unbalanced sample sizes among the inner and outer boundary plots (Anderson and Walsh 2013). We used the function betadisper for analysis of equal multivariate dispersion and the function adonis2 for PERMANOVA. The chosen dissimilarity measure for both procedures was Bray–Curtis dissimilarity.

To test the third hypothesis, we also used multivariate analysis. First, we checked the frequencies of the factor levels in each category and retained only those that constituted at least present in 10% for each level. As the general distribution of levels of boundary category levels was the same for CE and FM, this was done for the subsets together (Table 1). In the second step, the remaining categories were tested for homogenous in‐group dispersion. As this refers to species composition, it was tested for CE and FM separately. When homogenous in‐group dispersion for CE or FM was confirmed, the categories were tested for correlation with chi‐square test, and Cramer's V was used to determine the strengths of the correlations. If categories were correlated and the effect was greater than 0.3, we tested only the factor with a more equal distribution level. Each factor was tested separately with PERMANOVA for its significant influence on species composition. The categories that influence species composition were tested for their distribution on inner and outer boundaries to distinguish different drivers of the composition of the inner and outer CE and FM (Table 1).

The cover and height values of the crop and herbs were tested for correlations using the Spearman‐rank test. If the variables were correlated, we choose one of them for further analysis. The effect of the variables on the CE and FM vegetation were tested separately using PERMANOVA.

The mean values of the influencing cover and height variables were compared between inner and outer boundaries using the Wilcoxon rank test to identify possible differences in the drivers for the inner and outer boundaries (Table 1).

## 3 Results

### 3.1 Species and Species Numbers at the Inner and Outer Boundaries

**FIGURE 3:**
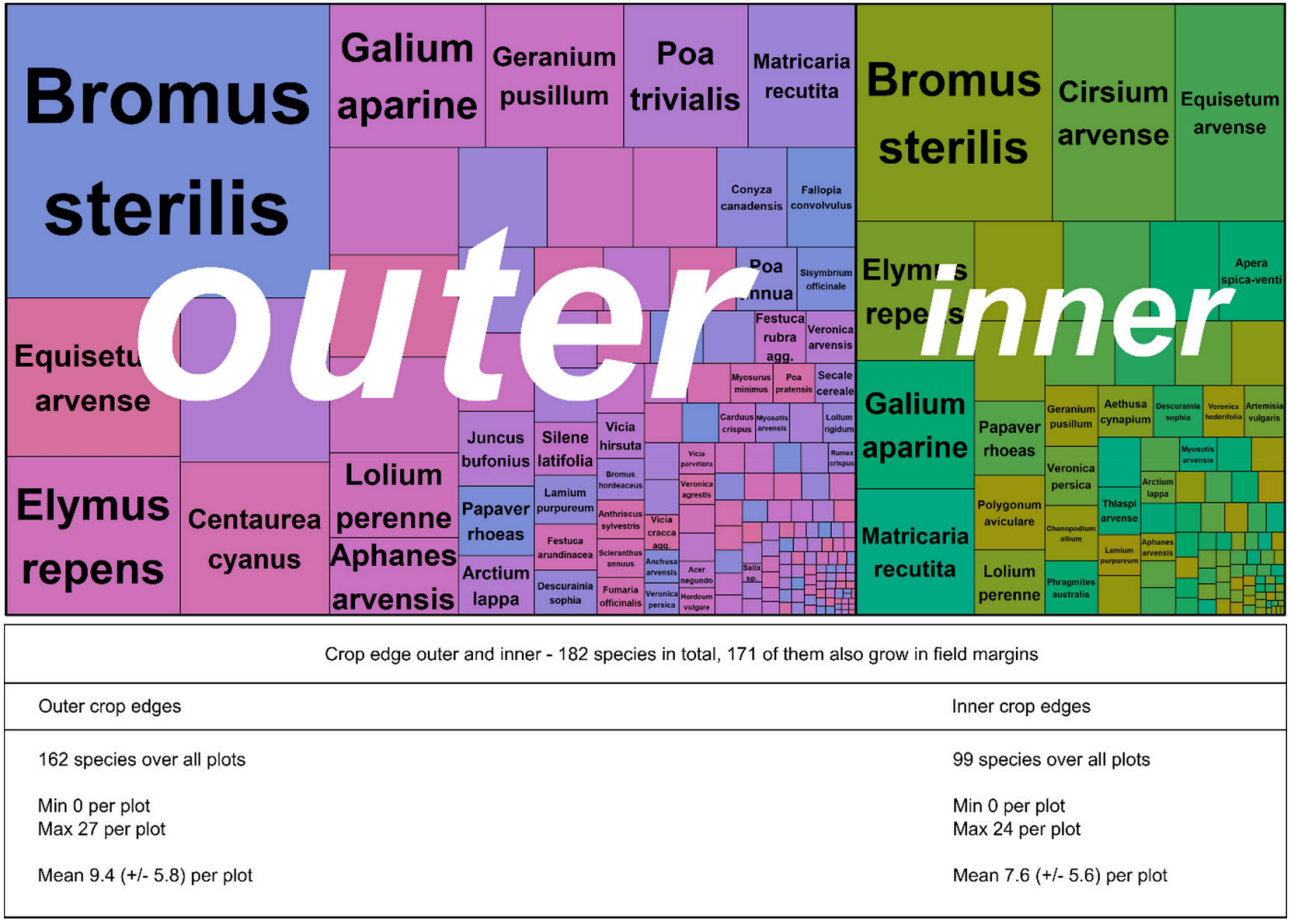
Treemap displaying species and their abundances on the outer and inner CEs. Table showing a summary of species numbers for the inner and outer CEs and both edges.

**FIGURE 4:**
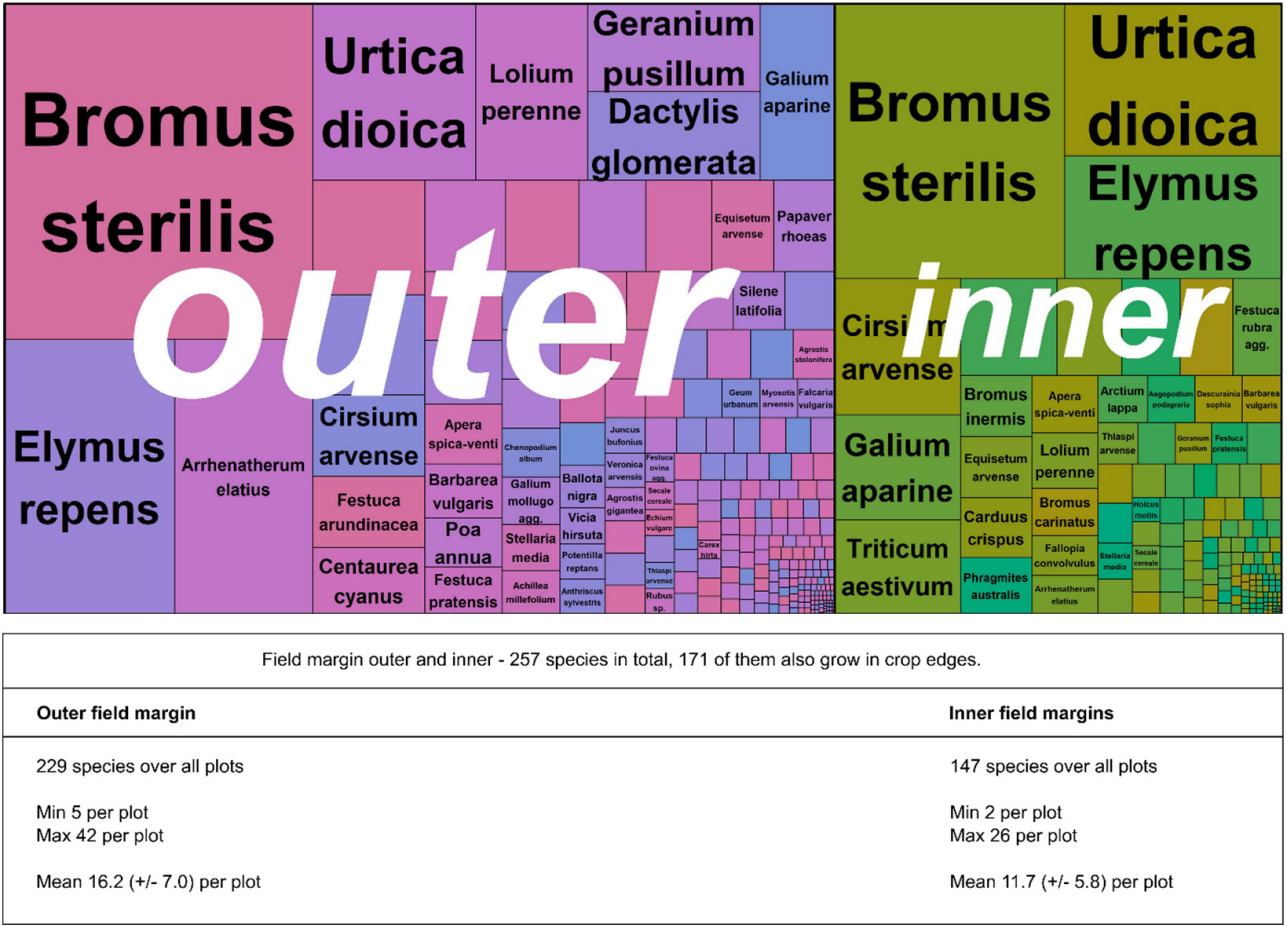
Treemap displaying species and their abundances on the outer and inner FMs. Table showing a summary of species numbers for the inner and outer FMs and both margins.

In total, we found 268 plant taxa in different types of field boundaries, including tree seedlings, shrubs and taxa that were identified only to genus level (as seedlings) (Figures 3 and 4).

The vegetation in the field boundaries was dominated by a few, mainly *Poaceae* species (Figures 3 and 4). In addition to grasses, typical species of ruderal and arable vegetation were found.

### 3.2 Species Diversity of the Inner and Outer Boundaries

**FIGURE A1:**
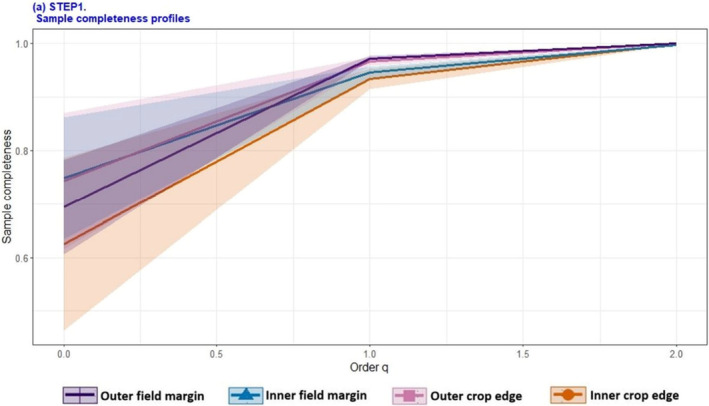
iNext4.steps—step 1—Sample completeness profiles of all subsets over the orders of *q* = (0, 1, 2).

With respect to species richness (*q* = 0), the sample completeness was between 0.63 for the inner CE and 0.75 for the inner FM (Figure A1), which indicates that approximately 25%–37% of species remain undetected (Table 2, step 1). The highest number of undetected species is assumed for the inner CE, and the lowest is assumed for the inner FM. Sampling completeness reaches a value of 1 when only the highly frequent species (*q* = 2) were considered, and values between 0.93 and 0.97 were observed for the Shannon diversity index‐ equivalent, when *q* was equal to 1 (Table 2).

**TABLE 2:** Results of the iNext.4steps procedure. Step 1: Sample completeness, step 2.1 and 2.2: Asymptotic analysis of species diversity, step 3: Coverage‐based diversity (non‐asymptotic approach), and step 4: Evenness. All the numbers are given for the orders of *q* = (0, 1, 2) and for all the assemblages: Inner CE, inner FM, outer CE and outer FM.

Completeness (step 1)						
	*q* = 0		*q* = 1		*q* = 2	
	Estimated	Undetected	Estimated	Undetected	Estimated	Undetected
Inner CE	0.63	0.37	0.93	0.07	1.00	0.00
Inner FM	0.75	0.25	0.95	0.05	1.00	0.00
Outer CE	0.74	0.26	0.97	0.03	1.00	0.00
Outer FM	0.66	0.31	0.97	0.03	1.00	0.00
**Asymptotic analysis (steps 2.1 and 2.2)**						
		*q* = 0 All species	*q* = 1 Abundant species		*q* = 2 Dominant species	
**Inner CE**						
Empirical (detected)		99.0	45.7		28.1	
Asymptotic (estimated)		158.3	52.3		29.1	
Undetected (via completeness)		59.3	6.5		0.9	
**Inner FM**						
Empirical (detected)		147.0	64.8		37.3	
Asymptotic (estimated)		196.5	72.4		38.4	
Undetected (via completeness)		49.5	7.7		1.1	
**Outer CE**						
Empirical (detected)		163.0	65.8		38.4	
Asymptotic (estimated)		218.2	71.1		39.2	
Undetected (via completeness)		56.2	5.3		0.8	
**Outer FM**						
Empirical (detected)		229.0	90.5		54.9	
Asymptotic (estimated)		329.8	96.6		55.7	
Undetected (via completeness)		100.8	6.1		0.8	
**Non‐asymptotic coverage‐based rarefaction and diversity (step 3)**						
**Maximum standardized coverage *C***_**max**_ **= 0.966**						
		*q* = 0 All species	*q* = 1 Abundant species		*q* = 2 Dominant species	
Inner CE		128.5	49.3		28.6	
Inner FM		166.3	67.5		37.7	
Outer CE		162.5	65.9		38.4	
Outer FM		212.6	89.2		54.6	
**Evenness (step 4)**						
Pielou J'						
		*q* = 0 All species	*q* = 1 Abundant species		*q* = 2 Dominant species	
Inner CE		0.80	0.38		0.22	
Inner FM		0.82	0.40		0.22	
Outer CE		0.82	0.40		0.23	
Outer FM		0.84	0.42		0.23	

**FIGURE 5:**
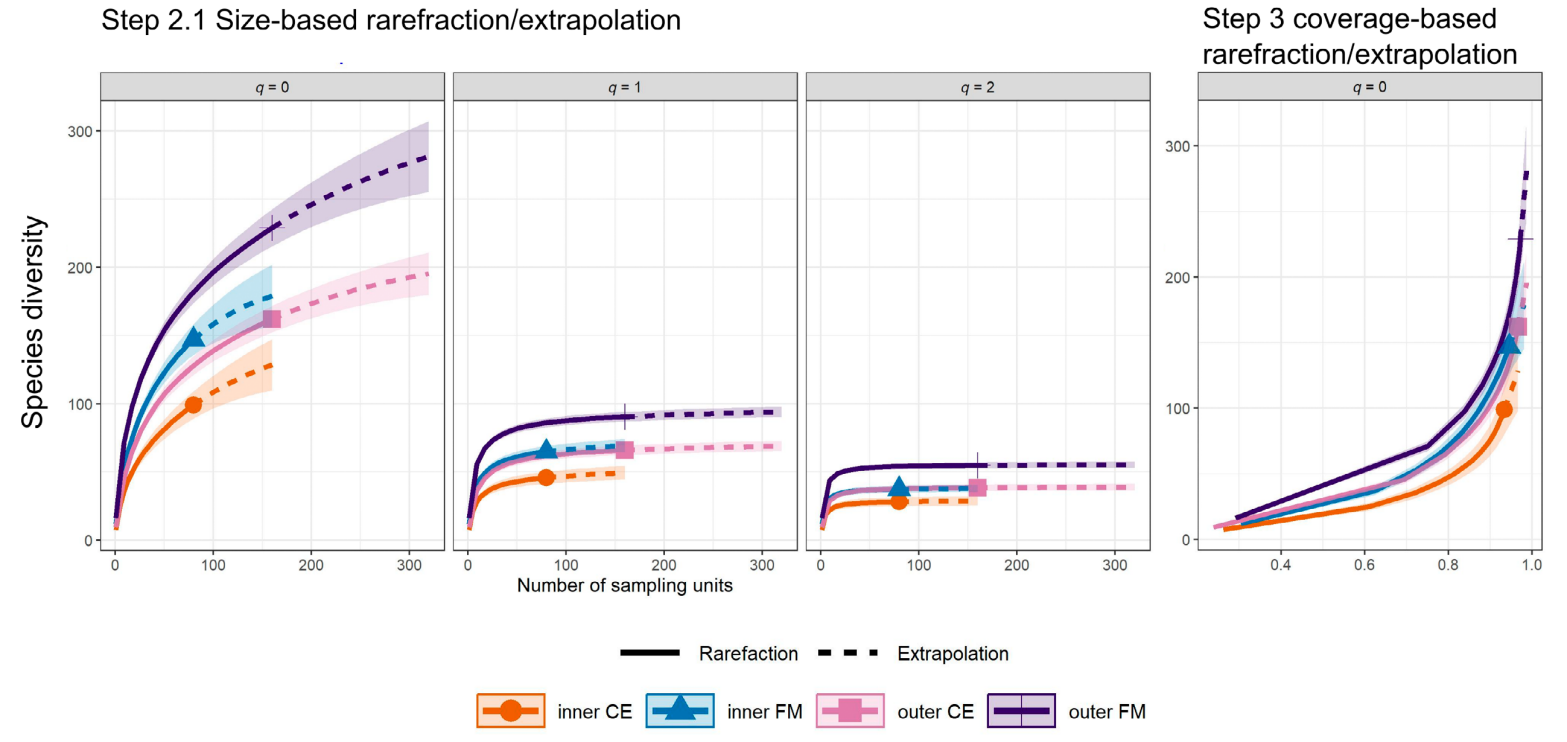
Asymptotic and non‐asymptotic diversity profiles. Species diversity for *q* = (0, 1, 2) plotted against the number of sampling units (asymptotic) and species diversity for *q* = 0 plotted against the sampling completeness (non‐asymptotic) for the assemblages of the inner and outer CE and the inner and outer FM.

For *q* = 1 and *q* = 2, the asymptotes of the species diversity curves in relation to the number of sampling units was reached after a steep increase for all four assemblages (Figure 5). To compare the number of species with the true diversities at each order of *q*, see Table 2.

In contrast to the results for the common and highly frequent species, the sampling was incomplete concerning species richness (*q* = 0), even when extrapolating up to double the sample size. The maximum coverage value at which all samples could be compared fairly is 0.96. The true diversity at this point was 128.5 species for the inner CE and 212.6 for the outer FM. The other two assemblages, that is, the inner FM and the outer CE included 166.3 and 162.5 species, respectively (Table 2).

**FIGURE A2:**
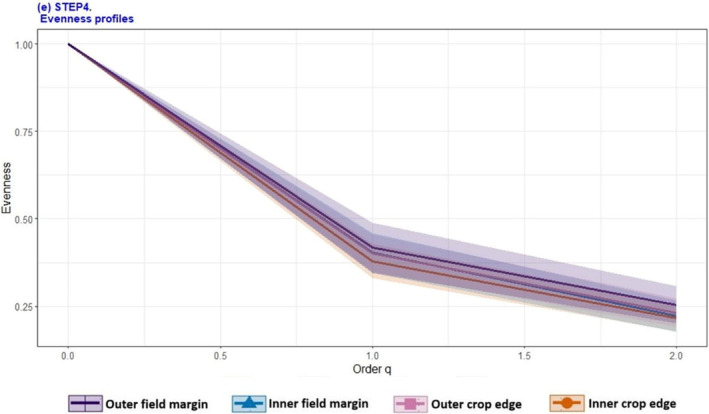
iNext4.steps—step 4—Evenness profiles of all subsets over the orders of *q* = (0, 1, 2).

The ranking of the assemblages revealed that the outer field margin always had the highest diversity, and the inner CE had the lowest diversity. The diversities of other assemblages were in between these values, and this finding was consistent across all the analysis steps used to calculate Hill diversity. As the confidence intervals of the diversities of the inner and outer FM and the inner and outer CE did not overlap (Figure 5), we concluded that there was a significant difference in the diversity of those communities. The evenness profile showed very similar evenness values over all the assemblages (Table 2 and Figure A2). At the orders *q* = 1 and *q* = 2, the values decreased, indicating few dominant species (Figure A2).

### 3.3 Species Compositions of the Inner and Outer Boundaries and Their Explanatory Factors

**FIGURE 6:**
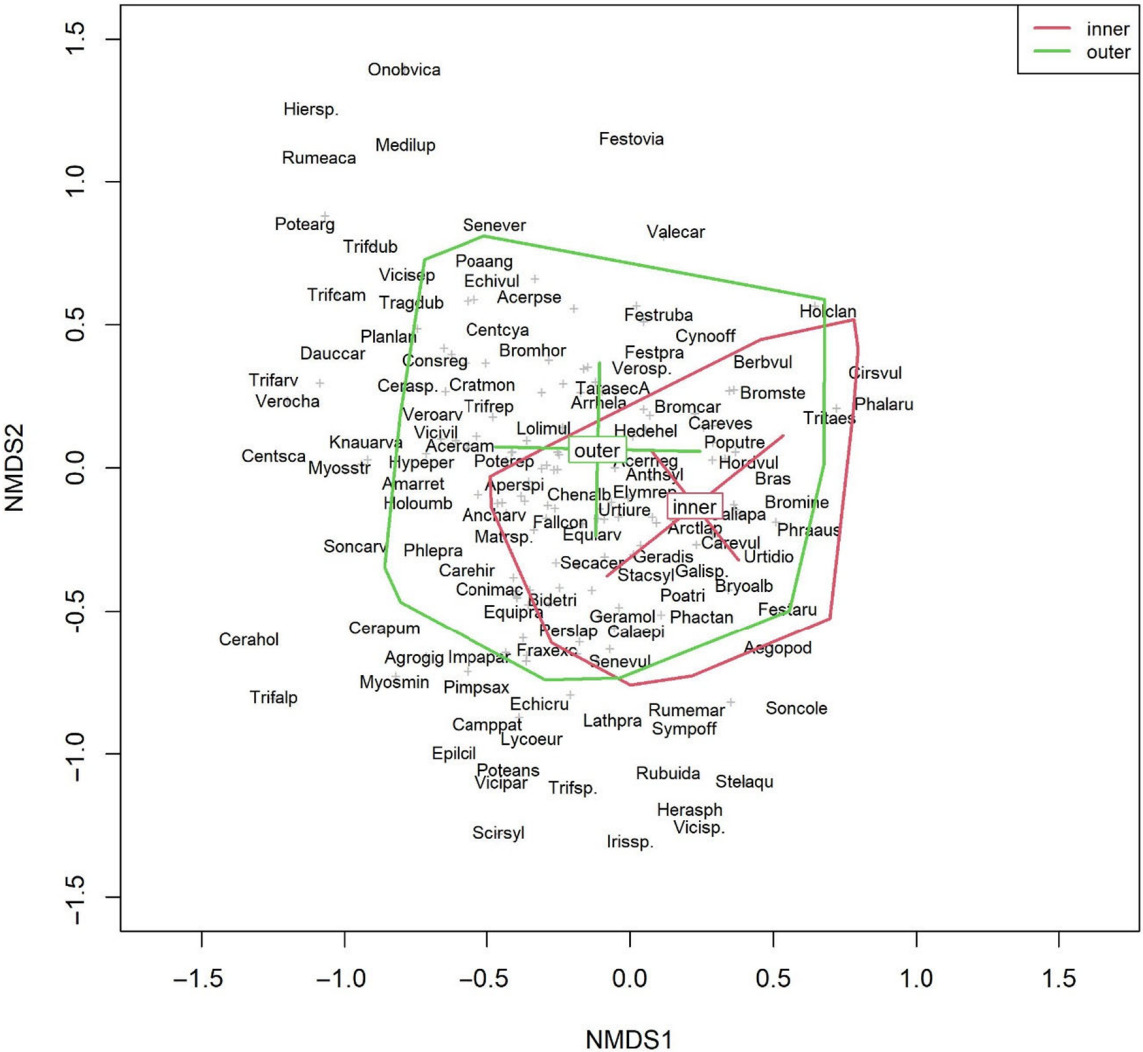
NMDS plot showing the species community of the FM and the centroids of the “inner” and “outer” FM groups. Names of the more dominant species are shown, and the remaining species are displayed as “+” symbols. Axes 1 and 2 are shown. Species names are abbreviated as follows: First four characters of the genus name, followed by the first three characters of the species name and the first character of the subspecies name, if needed. The hulls and centroids with bars were calculated based on standard deviations.

**FIGURE 7:**
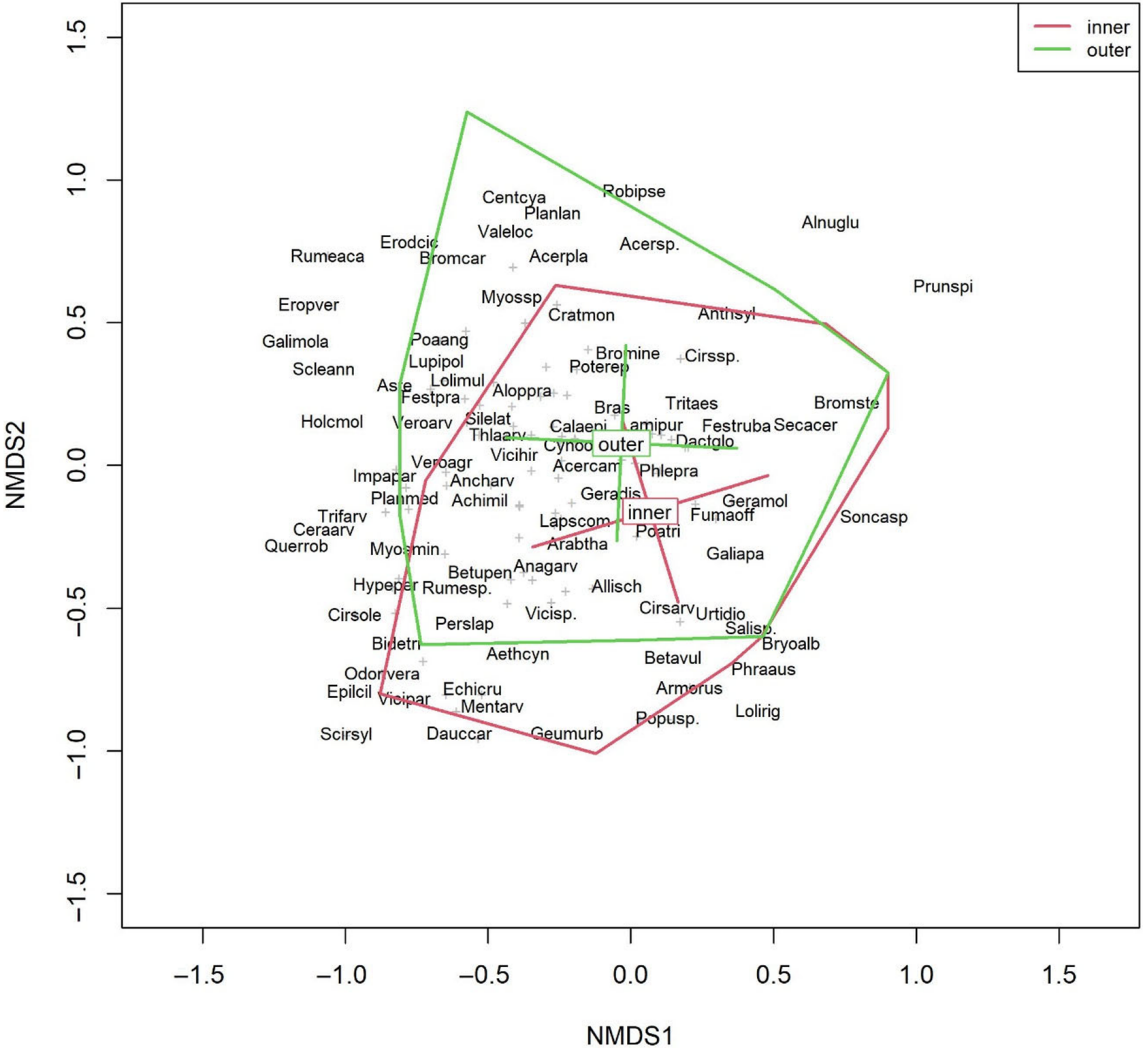
NMDS plot showing the species community of the CE and the centroids of the “inner” and “outer” CE groups. The names of the dominant species are shown, and the remaining species are displayed as “+” symbols. Axes 1 and 2 are shown. Species names are abbreviated as follows: First four characters of the genus name, followed by the first characters of the species name and the first character of the subspecies name, if needed. The hulls and centroids with bars were calculated based on the standard deviations.

The NMDS for the CE subset reached the best solution after 22 runs and obtained a stress value of 0.16. The NMDS for the FM subset obtained a stress value of 0.17 and the best solution after the maximum of 100 runs. The distinct centroids of the inner and outer FMs and CEs indicate that there were different species compositions for these groups. The group centroids of the inner and outer FMs were very clearly separated from each other (Figure 6). For the CE plots, the group centroids clearly differed on the second axis (Figure 7).

Within the FM subset, we saw a gradient from open, dry habitats to moist and shady habitats on axis 1. This gradient was reflected by species such as *Potentilla argentea*, *Scleranthus annuus*, and *Trifolium arvense* on the left side and species such as *Cirsium vulgare*, *Phragmites australis*, and *Urtica dioica* on the right side. In line with this gradient is the distinction between the outer and inner FM. The hulls of the inner FM were located on the right side, covering sites with moist and shady conditions. These sites were also partially covered by the outer margins, but their hull also stretched to the left, indicating a broader range of conditions in the outer FM (Figure 6).

To a lesser extent, this moisture gradient was also seen for the CE communities, here displayed on the second axis, with species such as *Bidens tripartita*, *Cirsium oleraceum* and *Phragmites australis* serving as indicators for moist sites at the bottom of the graph and *Centaurea cyanus*, *Erodium cicutarium* and *Rumex acetosella* serving as indicators for dry sites at the top of the graph (Figure 7). Analogous to the field margins, this was depicted by the hulls for the inner and outer CEs, although for the CEs, the hulls overlapped far more and showed less separation, indicating more similar communities between the inner and outer CE and a greater species overlap of these two communities. The observed difference between the centroids for the inner and outer CE and FM was tested with PERMANOVA (Table 3). Homogenous in‐group dispersion was given for the CE subset as well as for FM. For both subsets, the centroids of the groups have significantly different locations, with *p* values of 0.001. However, the *R* ^2^ values, which represent the amount of variation explained in relation to overall variation in the data, were low for both subsets: 0.014 for CE and 0.023 for FM.

**TABLE 3:** Boundary categories and cover and height values selected for PERMANOVA, tests for homogenous in‐group dispersion, Chi square and Spearman‐rank tests for correlations and PERMANOVA results. Levels of significance: ***p<0.001; **p<0.01; *p*<0.05. All *p* values smaller than 0.05 are marked bold.

Crop edge subset (CE)					
Category/variable	Hom. in‐group dispersion	Correlated with	Cramers V	Chi square *p* value	PERMANOVA *R* ^2^ and *p* value
**Pos_Field**	**Yes**	—	—	—	***R*** ^**2**^ = 0.014, *p* = 0.001***
Mowing	Yes	Road	0.37	0.000	—
**Road**	Yes	Mowing Water	0.37 0.25	0.000*** 0.000***	***R*** ^**2**^ = 0.012, *p* = 0.002**
**Water**	Yes	Road Trees	0.25 0.2	0.0000*** 0.000***	***R*** ^**2**^ = 0.007, *p* = 0.016*
**Trees**	Yes	Water	0.2	0.000***	***R*** ^**2**^ = 0.009, *p* = 0.007**
Hedge	Yes	Trees	0.14	0.040*	*R* ^2^ = 0.004, *p* = 0.578
			**Spearman‐rank corr. coefficient**	**Spearman‐rank *p* value**	
**Cov_herbs**	—	Cov_crop Herb_high	−0.451 0.542	0.000*** 0.000***	***R*** ^**2**^ = 0.042, *p* = 0.001***
Herb_high	—	Cov_herbs	0.542	0.000***	
Cov_crop	—	Crop_high	0.345	0.000***	
**Crop_high**	—	Cov_crop	0.345	0.000**	***R*** ^**2**^ = 0.015, *p* = 0.001***
**Field margin subset (FM)**					
**Pos_Field**	**Yes**	—	—	—	***R*** ^**2**^ = 0.03, *p* = 0.001***
Mowing	Yes	Road Trees	0.36 0.16	0.000*** 0.010**	
**Road**	Yes	Mowing Water	0.36 0.26	0.000** 0.000***	***R*** ^**2**^ = 0.03, *p* = 0.001***
**Water**	Yes	Road Trees	0.26 0.2	0.000*** 0.002**	***R*** ^**2**^ = 0.01, *p* = 0.004**
**Trees**	Yes	Mowing Water	0.16 0.2	0.010** 0.002**	***R*** ^**2**^ = 0.01, *p* = 0.016*
			**Spearman‐rank corr. coefficient**	**Spearman‐rank *p***	
**Herbs_high**	—	Cov_herbs	0.33	0.000***	***R*** ^**2**^ = 0.03, *p* = 0.001***
Cov_herbs	—	Herbs_high	0.33	0.000***	

The explanatory factors for the CE subset all had homogenous in‐group dispersion. Mowing was correlated with roads, with a Cramer's *V* value of 0.37, and was less frequent than roads, therefore we excluded mowing. The other categories were correlated with each other with Cramer's *V* values less than 0.3, and we tested their influence on species composition with PERMANOVA. Road, water, and trees significantly influence species composition on CEs, whereas hedge do not. The *R* ^2^ values were low, with roads having the highest value of 0.12. The cover and height values of the herbs and crops were also correlated. Consequently, we analyzed only herb cover and crop height. Both of these factors showed had a significant but low influence on species composition (Table 3). While crop cover was significantly greater in the inner CE, herb cover was not (Table 1).

For the FM subset we excluded hedges because of non‐homogenous in‐group dispersion. Roads, water and trees were correlated with Cramer's *V* values less than 0.3. Mowing was correlated with roads, with a Cramer's *V* value of 0.36 and was therefore excluded. Also in this subset, roads, trees and water significantly influenced the species composition with low *R* ^2^ values, and roads presented the highest *R* ^2^ value with 0.3 (Table 3). Crop cover and height were not recorded for FMs and herb cover and height were correlated. Therefore, we analyzed only herb cover for consistency with CE analysis. Herb covers had a small, but significant influence on species composition. The values for herb cover were significantly different between the inner and outer FMs (Table 1).

## 4 Discussion

For the first time, field boundary vegetation around habitat islands formed by kettle holes was described with respect to plant species, their diversity and their composition. Comparisons were made between the inner field boundaries and the outer field boundaries on the CE as well as on the FM. We found significant differences between the inner and outer field boundaries in the arable field (CE) and in the permanent vegetation (FM), which could be related to different explanatory variables.

### 4.1 Methods

The diversities of the four assemblages inner and outer CE and inner and outer FM were accurately described and compared using Hill diversity, calculated in the *iNext*.*4steps* package. Furthermore, the community composition and the differentiation between the inner and outer boundaries on both sides of the field border were demonstrated successfully with NMDS and PERMANOVA.

Disentangling the different explanatory factors for the compositions of the CE and FM required more analysis. The boundary categories were distributed irregularly between the inner and outer boundaries and correlated. Thus, we had to remove some of them from the analysis and address overlapping influencing factors.

The factors influencing the species composition were highly significant for all four subsets. However, the explained variation in the boundary categories and cover and height values was very low, ranging between 1% and 5%. This finding is in line with other studies trying to capture the factors influencing the species composition of plants, especially with large datasets (Le Coeur, Baudry, and Burel 1997; Lososová et al. 2004; Von Redwitz and Gerowitt 2018). The typical constrains of a field study design prevent us from testing for every variable we thought could have an impact. Crop type for example, representing a set of management decisions, could not be tested with PERMANOVA, because of a very unbalanced distribution of the crops. Nevertheless, we identified variables explaining the species composition, but they were not different between the CE and FM. Thus we have to reject hypothesis (iii), presuming different factors affecting the species composition of the CE and FM. Nevertheless, we identified factors that helped us to explain the difference in the species composition between the inner and outer CE and FM.

### 4.2 Species Richness

With this sampling design, we were able to effectively capture the dominant and common plant species in the field boundaries within the study region. Incompleteness in the species richness (*q* = 0) were observed for all four assemblages. According to Chao et al. (2020), it is typically more challenging to capture aspects of diversity with species richness, which is calculated via the number of singletons and doubletons, reaching its asymptote only when no more new species are found. The field boundaries around large fields are long and very heterogeneous at a very small spatial scale, thus, the chance of finding another species not already present in the sample remains high. This is illustrated by the diversity analysis.

Across all the aspects of the analyzed species diversity, the patterns of different field boundaries remained stable. The outer field margins always had the highest diversity and the inner CE had the lowest diversity. The inner FM and outer CE had very similar diversity values, across all orders of *q*. The parts of hypotheses (i) and (ii) concerning species richness can be accepted for the FM (the inner and outer FM differed significantly), but must be rejected for the CE (the inner and outer CE did not differ significantly).

As arable fields are subjected to seasonal interventions, the range of species that are able to grow in this environment successfully is limited relative to that in the FMs. Although many ruderal species are able to invade arable fields, at least within the first few meters, species numbers are often greater in FMs. Štefanić et al. (2020) reported 134 species in FMs, whereas 109 were found in the CEs. In general, the overlap of species in the first meter(s) of arable fields is high (Wietzke et al. 2020) but often unbalanced in favor of margins (Marshall and Arnold 1995; Aavik et al. 2008; Marshall 1989; Kiss et al. 1997). Accordingly, we found more species in margins and a high overlap of species, illustrating the character of the field boundary as a transitional zone (Marshall 1989).

Unlike the often species‐rich habitat islands themselves (Schöpke et al. 2019; Cousins 2006; Deák et al. 2020), the boundaries of kettle holes in our study presented significantly lower species diversities than the outer boundaries did. This pattern was observed in the CEs as well as in the permanent vegetation of the FMs.

Many species not present in the surrounding landscape matrix can be found inside kettle holes, with high turnover even in small areas (Schöpke et al. 2019). However, the heterogeneity observed in the interior of kettle holes seems to remain inside the kettle holes, whereas the areas within the arable field within the first meters from the field border are influenced by intensive agriculture in both directions. This was depicted by the lower species richness of inner borders.

Kettle holes are known to be threatened by the drift of fertilizer and pesticides (Vasić et al. 2020). The effect of this phenomenon might be even more severe in areas directly adjacent to arable fields, which, in the case of this study, is the surveyed area. German law dictates that not only kettle holes but also the banks of kettle holes are protected, however, the width of the protected area is not defined. This leads to uncertain borders of these landscape elements (Vasić et al. 2020) and might increase disturbance and input from the adjacent agricultural system.

Many studies of weed species diversity report much greater diversity at the edges of crop fields (Seifert et al. 2014; Batáry et al. 2017; Gabriel et al. 2006; Wietzke et al. 2020). Batáry et al. (2017) reported a decrease in plant species richness of approximately 25% from the CE to interior plots located 15 m into wheat fields, with almost no further decline to the center of the fields. In intensively managed agricultural landscapes, only a few species are found frequently in field interiors, and these species have both low abundances and low cover rates (Wietzke and Leuschner 2020; Wietzke et al. 2020; Seifert et al. 2014). In particular, in very large fields such as the ones examined in this study, the relatively low species richness on the inner CE may be related to field size and the inability of plants to cross these large distances (Fried et al. 2009). Thus, the lower species diversity at the inner boundaries depicts the general impoverishment of intensively managed arable fields.

Field size also affects the species richness of margin vegetation, as shown by Fried, Villers, and Porcher (2018), which could also explain the lower diversities in the inner field margins. Another possible explanation for the inner margins being less diverse could be the limited range of boundary structures in the inner field margins, at least for kettle holes. For other types of habitat islands, this might differ and depend on the characteristics of these biotopes. Species from bordering habitats such as forests, grasslands, and road verges are not present around kettle holes and thus cannot contribute to species diversity as they do for outer field margins.

### 4.3 Species Composition

With respect to the species composition, the field boundaries around the kettle holes were significantly different from the outer boundaries for both the CE as well and FM. While we hypothesized that different factors drive the species composition in the FM and CE, this could not be confirmed with our data. The boundary categories road, trees, and water influenced the species composition of both subsets, and herb cover also influenced the CE and FM. The only difference was the influence of crop height on the CE, but this was only recorded for the CE.

Trees and roads influenced the species composition of the field boundary vegetation in our study. Although these categories were correlated, we decided to test them both, because roads were found only near the outer boundaries, whereas the presence of trees was equally distributed between the inner and outer boundaries. Both categories therefore explain differences in species composition, but only the presence of roads can help explain the differences between the inner and outer FM and CE.

The other factor that differentiated the conditions and thus vegetation between the inner and outer boundaries was the presence of water directly around the plots. The presence of water was significantly greater for the inner boundaries. Consequently, we observed a moisture gradient, with moister conditions in the boundaries around the kettle holes. This is due to the nature of the kettle holes as small water bodies.

The influence of local site characteristics on the permanent vegetation of FM has been shown in many studies. In an Estonian study, the local boundary structure drove both, the species composition and the richness of plant species in the field margins, displaying a contrast between more dry and open habitats and shady and moist conditions near trees or ditches (Aavik and Liira 2010). Le Coeur, Baudry, and Burel (1997) reported a clear separation between field margin vegetation and the presence or absence of woody boundary elements. In a study on an Italian farm, the management practices applied next to the margins and in the margins mainly explained the species composition (Blaix and Moonen 2020). This study also found an effect of adjacent land‐use types, that is, the presence of trees or roads, on vegetation composition in margins.

The factors influencing the species composition of the boundaries in our study are consistent with those reported in other studies. We also found that trees, roads, and water drive species composition and create a moisture gradient, which can at least partly explain the differences between the inner and outer boundaries. Owing to the nature of kettle holes as habitat islands, the range of boundary structure categories was limited and the present categories reflect mostly shady or moist conditions. Additional open and dry habitats are found in the outer boundaries of fields and widen the range of possible species communities.

Interestingly, the factors influencing species composition in the CE were generally the same as those in the FM. The features directly surrounding the CE, namely, trees, roads, or water, also drive the species composition of the CE. Here again, the transition zone of the field boundaries becomes apparent.

We expected that management would have a strong influence on species richness as well as on species composition in the CE (Seifert, Leuschner, and Culmsee 2015; Fried, Norton, and Reboud 2008; Nagy et al. 2018). In our study, we tried to mitigate the differences between management practices by choosing only conventionally managed winter cereals. However, there remains heterogeneity at the local scale, because of the drift of inputs such as fertilizers or herbicides, and the reduced growth of crops due to shading or the activities of animals. These factors might create partly heterogeneous environmental conditions for other non‐crop plants growing on the crop edges (Seifert, Leuschner, and Culmsee 2015; Wietzke et al. 2020). We partly captured this heterogeneity through herb cover (CE and FM) and crop height (CE only), both of which influence species composition. Crop height (CE) and herb cover in FM differed significantly between the inner and outer boundaries. The inner boundaries have higher crop growth and crop cover values in the CE and higher herb cover and herb height values in the FM. This might be due to the previously mentioned higher moisture around the kettle holes. Additionally, the banks of kettle holes are often eutrophic (Vasić et al. 2020) and are sometimes also managed more intensively than the remaining fields to suppress weeds (personal communication with farmers).

High‐intensity agriculture is reflected in tall and dense crop stands, and these characteristics influence the non‐crop plants in the field (Wietzke et al. 2020; Seifert et al. 2014). This can lead to poorer species communities with a greater share of competitive grasses, which we can observe at inner field boundaries.

As kettle holes are a specific type of habitat islands, characterized by at least temporarily moist conditions and very heterogeneous manifestations (Kalettka and Rudat 2006; Pätzig et al. 2012), they cannot be easily compared with other types of habitat islands. Many studies address habitat islands represented by remnants of dry grasslands (Cousins and Lindborg 2008; Öckinger et al. 2012; Deák et al. 2020) although none of them describe the direct transition zone to bordering agricultural land use. In particular, the moisture gradient of the inner boundaries, observed in our study might not be transferable to other habitat islands.

## 5 Conclusion

We found that field boundaries around kettle holes in northeastern Germany had lower diversities and significantly different species compositions than boundaries on the outer field borders in the FM and CE. We expected that the local boundary structure would affect the permanent FM vegetation, which we confirmed with our results. Thus, the inherent characteristics of kettle holes are mirrored in the inner borders of FM vegetation, which have moist conditions and stronger growth by competitive plants. Surprisingly, despite the same field management practices on the outer and inner CE, the moister and more shaded conditions of the CE around the kettle holes were apparent in those communities. This is mitigated partly through increased crop cover and growth, lowering the growing conditions for non‐crop plants. Returning to the quotation by Wrzesien and Denisow (2016), who considered midfield islets as valuable sources of diversity in agricultural landscapes, we must conclude that diversity is lower at inner boundaries. Nevertheless, as we found different plant communities at inner boundaries, they might contribute to the overall diversity, especially in large fields.

## Author Contributions

**I. Heyer:** conceptualization (lead), formal analysis (lead), funding acquisition (lead), investigation (lead), methodology (lead), project administration (lead), visualization (lead), writing – original draft (lead), writing – review and editing (lead). **M. Wulf:** conceptualization (supporting), funding acquisition (supporting), methodology (supporting), project administration (supporting), supervision (lead), writing – review and editing (supporting).

## Conflicts of Interest

The authors declare no conflicts of interest.

### Open Research Badges

This article has earned Open Data, Open Materials and Preregistered Research Design badges. Data, materials and the preregistered design and analysis plan are available at [[insert provided URL(s) on the Open Research Disclosure Form]].

## Data Availability

Data and scripts are available at the BonaRes Repository under DOI: https://doi.org/10.4228/zalf‐ebfq‐0075.
